# Consequences of gaining an extra chromosome

**DOI:** 10.1007/s10577-023-09732-w

**Published:** 2023-08-25

**Authors:** Eduardo M. Torres

**Affiliations:** Department of Molecular, Cell and Cancer Biology, University of Massachusetts Chan Medical School, Worcester, MA 01605 USA

**Keywords:** Aneuploidy, Yeast, Human trisomy, Down syndrome, Nuclear morphology, Serine synthesis, Sphingolipids

## Abstract

Mistakes in chromosome segregation leading to aneuploidy are the primary cause of miscarriages in humans. Excluding sex chromosomes, viable aneuploidies in humans include trisomies of chromosomes 21, 18, or 13, which cause Down, Edwards, or Patau syndromes, respectively. While individuals with trisomy 18 or 13 die soon after birth, people with Down syndrome live to adulthood but have intellectual disabilities and are prone to multiple diseases. At the cellular level, mistakes in the segregation of a single chromosome leading to a cell losing a chromosome are lethal. In contrast, the cell that gains a chromosome can survive. Several studies support the hypothesis that gaining an extra copy of a chromosome causes gene-specific phenotypes and phenotypes independent of the identity of the genes encoded within that chromosome. The latter, referred to as aneuploidy-associated phenotypes, are the focus of this review. Among the conserved aneuploidy-associated phenotypes observed in yeast and human cells are lower viability, increased gene expression, increased protein synthesis and turnover, abnormal nuclear morphology, and altered metabolism. Notably, abnormal nuclear morphology of aneuploid cells is associated with increased metabolic demand for de novo synthesis of sphingolipids. These findings reveal important insights into the possible pathological role of aneuploidy in Down syndrome. Despite the adverse effects on cell physiology, aneuploidy is a hallmark of cancer cells. Understanding how aneuploidy affects cell physiology can reveal insights into the selective pressure that aneuploid cancer cells must overcome to support unlimited proliferation.

Aneuploidy is defined as the cellular state of having a chromosome number that is not an exact multiple of the haploid number. Understanding how aneuploidy affects cell physiology is important because the incidence of aneuploidy is associated with human disease and aging (Nagaoka et al. 2012; Ricke and van Deursen 2013). In addition, aneuploidy is a hallmark of cancer cells, and it has long been hypothesized that aneuploidy plays an active role in tumorigenesis (Boveri 2008). Recent studies suggest that the role of aneuploidy in tumor biology is complex and context-dependent (Ben-David and Amon 2020). The existence of other genomic alterations, including gene mutations, focal amplifications, chromosomal translocations, and chromosomal rearrangements promoted by chromothripsis, in addition to karyotypic heterogeneity caused by ongoing genomic instability and the complex tumor microenvironments depending on tumor type, can influence the role of aneuploidy in cancer (Burgess 2011, Banerji et al. 2012, George et al. 2015, Azizi et al. 2018, Shoshani et al. 2021, Steele et al. 2022).

The most common aneuploidy in humans that is compatible with life is the trisomy of autosome 21, which causes Down syndrome (cdc.gov). Aneuploidies involving sex chromosomes are also tolerated. An extra copy of the X chromosome in males causes Klinefelter’s syndrome (XXY), the most common aneuploidy after Down syndrome (Jacobs and Strong 1959; Los and Ford 2023). Females can also survive with three X chromosomes (Triple X syndrome) (Jacobs 1979; Otter et al. 2010). As for monosomies, females with a single X chromosome have Turner’s syndrome (Sybert and McCauley 2004). Aneuploidies involving the X chromosome are distinct from trisomies of autosomes because this chromosome is subject to dosage compensation mediated by a large non-coding RNA Xist (Loda et al. 2022). Xist transcriptionally silences one copy of the X chromosome in normal females, the extra X in males with Klinefelter syndrome, and two copies in triple X females (Schulz and Heard 2013). Similar mechanisms to inactivate and silence entire autosomes have not been reported. Remarkably, using genomic editing techniques introducing the Xist transgene in an autosome leads to the inactivation and silencing of that autosome (Lee and Jaenisch 1997; Jiang et al. 2013). Lastly, males with an extra copy of the Y chromosome (XYY syndrome) can also survive but show mild clinical symptoms (Bardsley et al. 2013). In this review, we summarize the data that supports the hypothesis that gaining a single copy of an autosome disrupts cellular homeostasis and that the deleterious effects of an extra chromosome increase proportionally with the number of protein-coding genes in that chromosome. We focused on studies of aneuploidy performed in the model organism yeast *Saccharomyces cerevisiae* and primary human cells. The conservation of phenotypes between these two eukaryotes highlights how aneuploidy affects conserved basic biological processes across different organisms independent of the identity of the genes on duplicated chromosomes.

## An extra chromosome lowers proliferation and viability

Aneuploidy is a problem of proteome imbalance as increasing protein-coding genes increases the deleterious effects on cell physiology. The yeast *S*. *cerevisiae* has been used as a model system to systematically assess the physiological consequences of gaining an extra copy of a single chromosome in the absence of other genomic alterations. Haploid yeast consists of 16 chromosomes, and using chromosome transfer techniques followed by selection, a series of isogenic aneuploid yeast strains, each harboring 17 chromosomes (1n + 1, referred to as disomes), were generated (Torres et al. 2007). Phenotypic characterization revealed that the disomes have lower proliferation rates relative to the euploid control. Lower cell viability and minor delays in the cell cycle, mainly during G1 and S phases, account for the proliferation defects of the disomes (Torres et al. 2007; Thorburn et al. 2013). Notably, the effects on viability and the delays in the cell cycle worsen with the size and number of open reading frames of the duplicated chromosome. Since yeast artificial chromosomes (YAC) as large as the yeast chromosomes without protein-coding genes do not cause similar defects, these results indicate that the adverse effects on proliferation increase with the number of extra genes (Torres et al. 2007).

The correlation between the number of genes on the extra chromosome and the loss of viability is not perfect in yeast. For example, an extra copy of chromosome VI, the second smallest chromosome, is not tolerated. Beta-tubulin *TUB2* and actin *ACT1* are on chromosome VI, and a single extra copy of either gene is toxic to the cell (Liu et al. 1992; Anders et al. 2009). In the opposite case, an extra copy of chromosome II which is the 7th largest chromosome in yeast shows minor proliferation defects. These two examples show that in addition to the cellular imbalance associated with aneuploidy, certain genes in the extra chromosomes can cause specific phenotypes. Indeed, gaining an extra copy of a given chromosome in yeast is a mechanism by which cells can adapt to mutations and environmental stress or become resistant to a particular drug (Hughes et al. 2000; Selmecki et al. 2006; Pavelka et al. 2010; Yona et al. 2012). Karyotypic analyses of wild yeast strains show various degrees of aneuploidy, suggesting that yeast is a microorganism that can exploit aneuploidy to adapt to multiple environments (Peter et al. 2018). Nonetheless, the systematic comparison of the euploid lab strain to isogenic strains with extra chromosomes under controlled growth conditions provides strong evidence of a fitness cost associated with gaining an extra chromosome. Consistently, aneuploid yeast strains with complex karyotypes recovered from the meiosis of triploids (3n) show growth defects compared to euploid controls under optimal growth conditions (Pavelka et al. 2010). Still, several aneuploids show better proliferation under specific cellular stresses. Cells tend to gain an extra copy of a chromosome that contains a gene that, when increased in copy number, helps alleviate the stress. Yet, when the stress is no longer present, yeast cells tend to lose the extra chromosome (Yona et al. 2012).

**Fig. 1:**
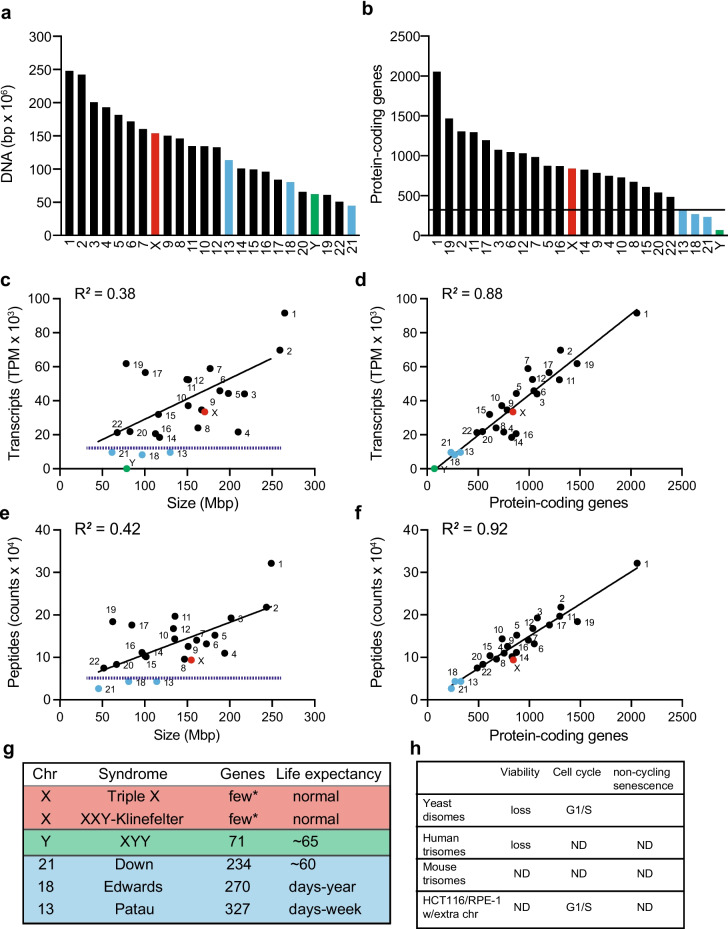
The number of protein-coding genes increases the deleterious effects on cell physiology. **a** Human chromosomes are arranged by DNA content. Data were obtained from Genome Reference Consortium (GRCh38.p14). **a–g** To highlight the data of autosomes 13, 18, and 21 are colored in light blue, and sex chromosomes X and Y in red and green, respectively. **b** Human chromosomes are arranged by the number of protein-coding genes. Autosomes 13, 18, and 21 are not the smallest in size but encode the least number of proteins compared to other autosomes. **c** Number of transcripts per million counts (TPM) per chromosome does not correlate with chromosome size. RNAseq of a euploid primary human fibroblast from Hwang et al. 2021 (Hwang et al. 2021) was used. Prism 9 software was used to calculate linear regression analysis. **d** Number of transcripts per million counts (TPM) per chromosome correlates with number of protein-coding genes. Prism 9 software was used to calculate linear regression analysis. **e** Number of peptide counts per chromosome do not correlate with chromosome size. Tandem mass tag (TMT) proteomics of a euploid primary human fibroblast from Hwang et al. (Hwang et al. 2021) was used. Prism 9 software was used to calculate linear regression analysis. No proteins encoded on chromosome Y were detected. **f** Number of peptide counts per chromosome correlates with the number of protein-coding genes. Prism 9 software was used to calculate linear regression analysis. **g** Summary of the number of genes expressed and life expectancy in human trisomies. Asterisk (*) = few genes in the extra copy of chromosome X escape transcriptional silencing by Xist (Disteche 1995). RNAseq analysis of human fibroblast from male donors detects only three genes expressed at low levels from chromosome Y. **h** Summary of the effects of different trisomies on viability, cell cycle, and senescence. Yeast results are from Torres et al. (Torres et al. 2007), human trisomies from Hwang et al. (Hwang et al. 2021), mouse trisomies from Williams et al. (Williams et al. 2008), and human cell lines from Stingele et al. (Stingele et al. 2012). ND, not detected

A significant correlation between the number of genes on the extra chromosome and the deleterious effects on cell viability is also observed in humans. Human autosomes are numbered by their relative size under the microscope, with chromosome 1 being the largest and chromosome 22 being the smallest. However, the number of protein-coding genes per chromosome does not correlate with chromosome size (Fig. 1a, b). Importantly, transcriptome analyses and quantitative proteomics validate that the number of transcripts and proteins per chromosome in the cell correlate with the number of open reading frames within the chromosomes and poorly correlate with the size of each chromosome (Fig. 1c–f). Only chromosomes 21, 18, and 13, which encode 234, 270, and 327 protein-coding genes, respectively, the least number of genes among human autosomes, lead to viable trisomies (2n + 1). In addition, the deleterious effects on development within these three trisomies also correlate with the number of genes. Individuals with Down syndrome can live to adulthood, while individuals with Edwards and Patau syndromes live from a few weeks up to a year (Fig. 1g) (Rasmussen et al. 2003). At the cellular level, analysis of the proliferation properties of primary human skin fibroblasts (HSFs) isolated from donors with Down, Edwards, or Patau syndromes shows that gaining an extra copy of chromosome 21, 18, or 13 causes proliferation defects compared to euploid controls (Fig. 1h) (Hwang et al. 2021). Like aneuploid yeast, trisomic HSFs show lower viability compared to euploid controls. However, early passage trisomic cells do not show delays in a particular stage of the cell cycle or senescence as assayed by beta-galactosidase staining.

Another approach to investigate the effects of gaining an extra chromosome on cell physiology includes the isolation of mouse embryonic fibroblasts (MEFs) with an extra chromosome (Williams et al. 2008). These cells are recovered from embryos of crosses between mice that harbor Robertsonian translocations. As in yeast and humans, trisomic MEFs for chromosomes 1, 13, 16, or 19 show proliferation defects compared to the euploid controls (Williams et al. 2008). Although no trisomy leads to a viable mouse, there is also a correlation between the survival of a trisomic embryo and the size of the extra chromosome; that is, the larger the extra chromosome, the earlier the embryo dies (Dyban and Baranov 1987; Torres et al. 2008). Intriguingly, despite their poor proliferation, trisomic MEFs show no cell cycle delays, loss of viability, or increased senescence. One possibility is that the assays utilized were not sensitive enough to detect minor defects in these processes.

Even in the context of other genomic alterations, including cancer cell lines, an extra copy of a chromosome usually hampers proliferation. An additional copy of chromosome 3 or 5 was introduced into HCT116 cells via micronuclei-mediated chromosome transfer (Stingele et al. 2012). HCT116 is a colon cancer cell line that harbors amplified regions of chromosomes 8, 10, and 17 and loss of chromosome Y in addition to mutations in the ras proto-oncogene. In the same studies, extra copies of chromosomes 5 and 12, or 21, were introduced in RPE1, a human telomerase reverse transcriptase-immortalized epithelial cell line that harbors an extra copy of chromosome 10. A separate study introduced an extra copy of chromosome 13 in DLD1, a pseudo diploid colorectal adenocarcinoma cell line with mutations in Kras, PI3KCA, and TP53 (Upender et al. 2004). Because the original genomic alterations are present in the recovered cell lines with the extra chromosome, these approaches allow for assessing the effect of adding an extra chromosome relative to the original karyotype. These studies showed that introducing an extra chromosome in HCT116, RPE1, or DLD1 cells hampers proliferation. In HCT116 and RPE1, an extra chromosome causes cell cycle delays, mainly in G1/S, with no noticeable changes in viability or cell senescence. The lack of cell death could be because these cell lines can better tolerate aneuploidy than primary cells due to their original altered karyotype. In the case of HCT116, tetrasomy (2n + 2) for chromosomes 3 or 5 was recovered. Tetrasomic cell lines show worse proliferation defects than the trisomy counterparts supporting the hypothesis that the increasing number of genes correlates with the degree by which cell proliferation is affected. Lastly, targeting the spindle assembly checkpoint in RPE1 cells causes chromosome missegregation and increases aneuploidy (Santaguida et al. 2017). Analysis of the growth properties of the recovered aneuploids shows that cells with complex karyotypes involving the loss or gain of 3 or more chromosomes do not divide and arrest at G1. In contrast, cells with lower karyotype complexity involving the gain or loss of 1 or 2 chromosomes can continue dividing, yet they show cell cycle defects during S and M phases (Santaguida et al. 2017, Garribba et al. 2023).

Extra DNA alone does not cause similar phenotypes as increasing protein-coding genes. As mentioned above, yeast harboring artificial chromosomes that do not encode protein-coding genes do not show significant phenotypes compared to disomic strains. In humans, aneuploidies associated with the X chromosome are viable and cause mild clinical symptoms because Xist silences the extra X. X is the 8th largest human chromosome, larger than 13, 18, or 21, suggesting the presence of excess DNA material in the cell does not cause significant phenotypes. In addition, the phenotypes associated with an extra copy of Y in XYY syndrome are not as severe as in Down syndrome despite this chromosome being larger than chromosome 21 (Bardsley et al. 2013; Antonarakis et al. 2020). An additional copy of Y may not be as deleterious as other chromosomes because it encodes the least protein-coding genes (~ 70) among all human chromosomes, and these genes are mainly expressed during male development and specifically expressed in the testis with only a handful of genes expressed in somatic tissues (Godfrey et al. 2020).

In summary, slow cell cycle progression through G1 and S phases and lower viability account for the slow proliferation of aneuploid yeast and human cells (Fig. 1h). The mechanisms that delay entry into the cell cycle remain to be determined. The slow synthesis of cyclins accounts for some of the effects in yeast, but how an extra chromosome causes these delays is poorly understood. Loss of cellular viability may be associated with the disruption of the morphology and integrity of the nucleus (see below). Several other studies support the hypothesis that the degree of deleterious effects of an extra chromosome correlates with the number of genes in other organisms. Seminal studies in plants show a correlation between the size of the extra chromosome and the effects on fitness (Blakeslee 1921; McClintock 1929). Although there are no viable trisomies in *Drosophila* or *C*. *elegans*, there is a correlation between the size of the DNA in partial trisomies and fitness defects (Lindsley et al. 1972; Hodgkin 2005). These studies support the hypothesis that in addition to the increased activity of a particular gene on the extra chromosome, there is a common cellular consequence to aneuploidy: decreased cellular fitness, which deteriorates with increasing protein-coding genes in the duplicated chromosome.

## Transcript levels increase proportionally to gene copy number without evidence of gene dosage compensation

**Fig. 2:**
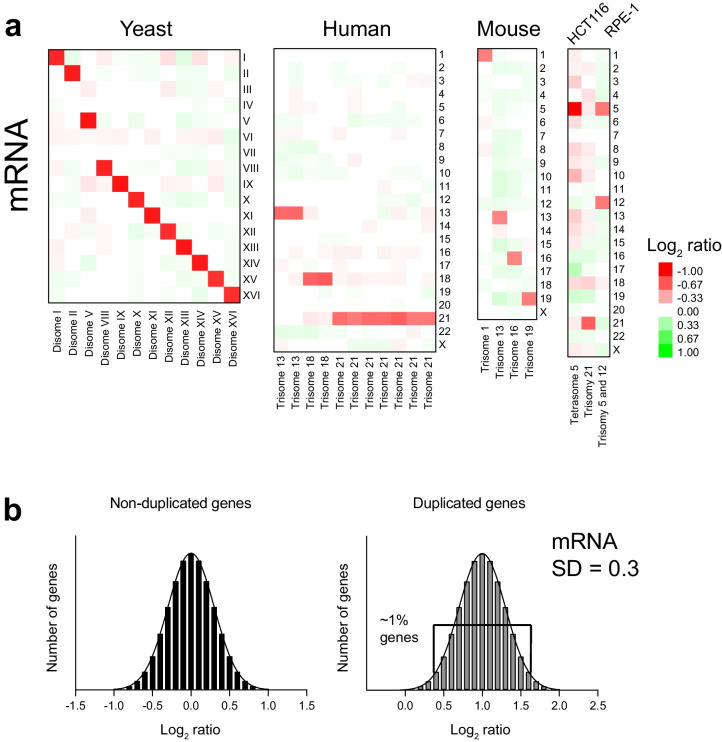
Transcript levels increase proportionally to gene copy number in aneuploid cells. **a** Heat map of the average log_2_ ratio of gene expression per chromosome in aneuploid cells relative to controls. Yeast expression data was obtained from Torres et al. (Torres et al. 2007), human trisomies from Hwang et al. (Hwang et al. 2021), mouse trisomies from Williams et al. (Williams et al. 2008), and human cell lines from Stingele et al. (Stingele et al. 2012). **b** Histograms of the log_2_ ratios of gene expression of non-duplicated (left, mean = 0) and duplicated genes (right, mean = 1) with a standard deviation (SD) of 0.3 as measured in aneuploid yeast strains. Plots indicate that only a handful of genes, 1.45%, fall outside 2 * SD from the mean (*p* < 0.05). If a particular gene shows a log_2_ ratio lower than the expected value of 1 but higher than 0.4, it cannot be considered as dosage compensated because it falls within the expected variability of population measurements with a *p* value higher than 0.05

Gene expression profiles of disomic yeast strains revealed that the mRNA expression levels of the genes encoded in the extra chromosome increase proportionally with gene copy number (Torres et al. 2007). On average, the mRNA levels of duplicated chromosomes increase the predicted twofold relative to the euploid control (log_2_ ratio (2n/1n) = 1, Fig. 2a). The same results have been obtained in the analyses of gene expression profiles of 5 aneuploid yeast strains harboring more than one extra chromosome isolated from triploid meiosis (Pavelka et al. 2010). HSFs trisomic for chromosomes 21, 18, or 13 and MEFs trisomic for chromosomes 1, 13, 16, or 19 show, on average, the predicted 1.5-fold (log_2_ ratio (3/2) = 0.6, Fig. 2a) increased expression of the trisomic chromosomes (Williams et al. 2008, Hwang et al. 2017). A proportional increase in gene expression of trisomic chromosomes is also seen in HCT116, RPE1, and DLD1 cell lines, despite epigenetic abnormalities induced by the micronucleation step to transfer them into cells (Fig. 2a) (Upender et al. 2004; Stingele et al. 2012; Agustinus et al. 2023). Therefore, a conserved consequence of gaining an extra chromosome in yeast and humans is the proportionally increased transcription of the different chromosomes without evidence of dosage compensation at the chromosome level. Unlike the X chromosome, which is silenced by Xist, mechanisms to silence autosomes do not appear to exist in human cells.

Numerous studies paint a complex relationship between gene copy number and changes in gene expression, fueling the debate whether the amounts of mRNA of duplicated chromosomes do or do not proportionally scale with copy number in yeast and human samples (Kojima and Cimini 2019). In some cases, there is a disagreement on the methods to analyze transcript data and to define a cutoff for a gene to be considered dosage compensated (Hose et al. 2015, Torres et al. 2016, Hwang et al. 2021, Hunter et al. 2023). An important point to consider is that genome-wide measurements of mRNA levels of single genes fall within a normal distribution (Fig. 2b). As expected, each set of measurements shows a given standard deviation arising from experimental and biological variability. The variability when measuring DNA copy numbers between two samples is small because the amount of DNA in each sample is similar. However, comparing RNA molecules between samples is much noisier as transcripts can vary several orders of magnitude depending on how much a particular gene is expressed in each cell type. Other factors influencing gene expression include cell growth conditions and karyotype heterogeneity due to genomic instability. In the yeast disomes, where the presence of the extra chromosome is selected for, and the population of cells is 99% of the predicted karyotype as analyzed by comparative genome hybridization, the standard deviations of mRNA of duplicated genes are in the order of log_2_ ratios of 0.3 (Fig. 2b) (Torres et al. 2007, Dephoure et al. 2014). There are a few genes (~ 1%) in the tails of the distribution, 2 standard deviations away from the mean of 1, that constitute outliers expected from biological measurements (Fig. 2b). In the case of human trisomy, the detection range is smaller as the mean log_2_ ratio of trisomy genes is the expected 0.6, and the standard deviation of the distribution is 0.3 (Hwang et al. 2021). Two standard deviations from the mean (*p* values < 0.05) fall below a log_2_ ratio of 0. Therefore, genome-wide measurements alone may not be sufficient to determine if a duplicated gene is being compensated at the transcriptional level. Other factors contributing to values lower than expected include the methodology to normalize the data, sample homogeneity, and interindividual variability (Hwang et al. 2021, Hunter et al. 2023). Concerning genomic instability, if part of the population of cells is losing or gaining chromosomes, genome-wide measurements of a mixed population of cells can lead to the incorrect interpretation that dosage compensation of a duplicated chromosome is taking place (Sheltzer et al. 2011, Torres et al. 2016). The fact that aneuploidy causes genomic instability raises technical issues to firmly conclude that a specific gene in a duplicated autosome is subject to dosage compensation using genome-wide approaches.

Analyses of the transcriptome profiles of yeast and human cells have not identified a conserved and specific transcriptional signature in response to the presence of an extra chromosome. Instead, most differentially expressed genes other than the duplicated genes in aneuploid cells are associated with slow proliferation (Torres et al. 2007; Sheltzer et al. 2012). In addition to the increased expression of the duplicated genes, disomic genes show a gene signature coined the environmental stress response, which is caused by slow growth (Gasch et al. 2000; Torres et al. 2007; Slavov et al. 2012). Analysis of the gene expression profiles of aneuploid yeast recovered from triploid meiosis revealed that 2 of 5 strains do not show similar gene responses, suggesting that the stress signature is not an obligate response to aneuploidy (Pavelka et al. 2010). Analysis of trisomic MEFs or HSFs gene expression profiles did not reveal a common set of genes differentially expressed among the different aneuploids (Williams et al. 2008, Hwang et al. 2021). In addition, analyses of the gene expression signature in trisomy 21 cells have led to several publications, each describing a different set of genes associated with various processes without reaching a consensus (Letourneau et al. 2014, Sullivan et al. 2016, Zhu et al. 2019, Hwang et al. 2021). Whether a transcriptional signature associated with a specific cellular process in response to the presence of an extra chromosome is conserved in eukaryotes across different cell types remains to be established. One possibility is that a transcriptional response to aneuploidy may depend on the cell type analyzed.

## An extra chromosome causes a proportional increase in protein levels, except for subunits of multiprotein complexes

**Fig. 3:**
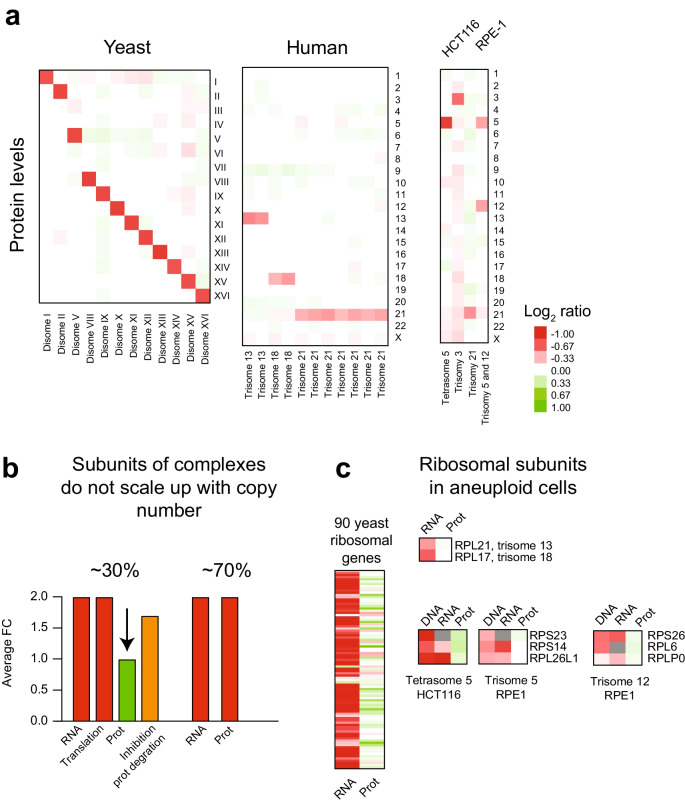
Protein levels increase proportionally to gene copy number in aneuploid cells, except for subunits of multiprotein complexes. **a** Heatmap of the average log_2_ ratio of protein levels per chromosome in aneuploid cells relative to controls. Proteomics data for yeast was obtained from Dephoure et al. (Dephoure et al. 2014), human trisomies from Hwang et al. (Hwang et al. 2021), and human cell lines from Stingele et al. (Stingele et al. 2012). **b** About 30% of duplicated proteins enriched for subunits of multiprotein complexes do not scale up with gene copy numbers in yeast and human aneuploid cells (green bar). Ribosome footprinting in yeast disomes shows that the attenuated proteins are translated proportionally to gene copy number. Inhibition of protein degradation leads to increased levels of the attenuated proteins within 90 s (orange bar). **c** Heatmaps of mRNA levels and protein levels of duplicated ribosomal subunits in yeast and aneuploid human cell lines are shown. This gene set invariably shows increased transcript levels, but the proteins are degraded—color scale bar of log_2_ ratio is the same as in **a**

Quantitative proteomics revealed that the protein levels of the duplicated genes in disomic yeast strains and trisomic HSFs for chromosomes 21, 18, or 13 increase with gene copy numbers (Fig. 3a) (Dephoure et al. 2014, Hwang et al. 2021). However, these measurements show that only ~ 70% of duplicated genes lead to a proportional twofold and 1.5-fold increase in protein levels in disomic yeast and trisomic HSFs, respectively. Unlike the RNA measurements that fit a normal distribution, protein measurements show significant skewness toward lower values than expected. The proteins that do not proportionally increase with copy number are enriched for subunits of macromolecular complexes. Similar results were observed in the HCT116 and RPE1 cell lines with extra chromosomes (Fig. 3a) (Stingele et al. 2012). These results support the hypothesis that a conserved consequence of aneuploidy is increased translation of the genes present in the extra chromosome with a substantial number of proteins enriched for subunits of multiprotein complexes being attenuated.

Ribosome footprint profiles of translation of aneuploid yeast showed that the duplicated genes, including subunits of complexes, are being translated with the predicted twofold increase in ribosomal occupancy (Dephoure et al. 2014, Taggart and Li 2018). These results indicate that increased translation of some duplicated genes does not yield the expected proportional increase in protein levels, most likely due to protein degradation. In support of this hypothesis, quantitative proteomics of disomic yeast strains treated with proteasome and autophagy inhibitors led to the accumulation and increased levels of the attenuated proteins within 90 s (Fig. 3b) (Dephoure et al. 2014). The ribosome subunits are a set of genes that show increased transcript levels when duplicated. Still, their protein levels do not increase with copy number in aneuploid yeast and human cells (Fig. 3c). Cells have evolved complex mechanisms to ensure equal expression levels of subunits of multiprotein complexes despite being encoded in the different parts of the genome (Li et al. 2014; Taggart and Li 2018). However, when a chromosome is gained, these mechanisms cannot compensate for the extra copy of the subunits encoded on the additional chromosome leading to increase transcription and translation (Taggart and Li 2018). Increased protein levels of one subunit are not coupled with the upregulation for the whole multiprotein complex, and unassembled subunits are, therefore, unstable and rapidly degraded. Consequently, gaining an extra chromosome leads to increased translation, folding of stable proteins, and degradation of a subset of unstable proteins, most of which are subunits of macromolecular complexes. It is important to distinguish between the compensation of protein levels by increased degradation of duplicated proteins in aneuploid cells and dosage compensation mediated by transcriptional silencing. Degradation of excess proteins is coupled to increases in metabolic demand associated with increased transcription, translation, protein folding, and protein degradation, all energy-costly cellular processes. Still, whether a few duplicated genes exist that elicit specific mechanisms that induce their compensation at the transcriptional level remains to be determined.

## An extra chromosome causes proteotoxic stress

Increased protein synthesis causes proteotoxic stress in aneuploid cells. Disomic yeasts are hypersensitive to drugs that inhibit the proteasome and protein folding and accumulate protein aggregates positive for the chaperone Hsp104 (Torres et al. 2007; Oromendia et al. 2012). Biochemical purification and quantification of protein aggregates in aneuploid yeasts revealed that proteins expressed for the extra chromosome are enriched in these aggregates (Brennan et al. 2019). Enrichment of the proteins of duplicated genes into aggregates is thought to help sequester them away to alleviate the toxicity associated with increased protein levels. Still, the mechanism for the enrichment of duplicated proteins in aggregates and whether the protein aggregates are detrimental to the cell are unclear. In MEFs, trisomies for chromosomes 1, 13, 16, or 19 increase sensitivity to compounds that induce proteotoxic stress, have higher basal levels of autophagy, and accumulate increased amounts of the chaperone HSP72 (Tang et al. 2011). Thus far, whether HSFs trisomic for chromosomes 13, 18, or 21 have identical phenotypes is not established. An interesting possibility is that the trisomies of these chromosomes are viable because the relatively small number of genes does not cause as severe proteotoxic stress as the larger chromosomes. Chromosomes 13, 18, and 21 encode 234, 270, and 327 protein-coding genes, representing 1.15%, 1.32%, and 1.60% of 20,399 human proteins. In the case of the trisomic MEFs, chromosomes 1, 13, 16, and 19 encode 1275, 899, 724, and 734 genes representing 5.52%, 3.89%, 3.13%, and 3.18% of 23,099 protein-coding genes. These observations suggest that there might be a threshold for the number of extra proteins (greater than 1.6% of the total protein) required to induce significant proteotoxic stress. Indeed, quantifying protein aggregates of RPE1 cells harboring an extra copy of chromosome 21 did not show enrichment of proteins encoded on 21 in purified aggregates. In contrast, cells with an extra copy of chromosome 12 did show such enrichments (Brennan et al. 2019). Similarly, an extra copy of chromosome I, one of the smallest duplicated chromosomes analyzed, failed to induce significant phenotypes associated with proteotoxic stress in yeast. A possible explanation for the tolerance of trisomy 21 in humans may be that the small number of protein-coding genes do not cause proteotoxic stress as other chromosomes. Lastly, HCT116 and RPE1 harboring extra chromosomes show a series of phenotypes consistent with higher proteotoxic stress, including accumulation of foci positive for ubiquitin, increased sensitivity to an HSP90 inhibitor, and upregulation of autophagy pathways (Stingele et al. 2012; Donnelly et al. 2014). Increased LC3 lipidation, LC3 foci, mRNA and protein levels of the autophagy adaptor sequestosome 1, p62, coupled with a normal turnover of fluorescently labeled LC3, led to the conclusion that an extra chromosome activates autophagy (Stingele et al. 2012). Interestingly, autophagy pathways are overwhelmed as an immediate response following chromosome missegregation in RPE1 cells, evidenced by the accumulation of autophagosomal proteins within lysosomes (Santaguida et al. 2015). This result suggests that gaining a chromosome causes selective pressure in cells to upregulate autophagy to tolerate extra chromosomes eventually.

Further evidence that aneuploidy causes proteotoxic stress comes from the unbiased identification of mutations that improve or lower the fitness of aneuploid cells. Spontaneous mutations in yeast revealed that the loss of function of the deubiquitinating enzyme *UBP6* (*ubp6∆*) minimally impacts the fitness of euploid cells but improves the fitness of most disomic strains (Torres et al. 2010). Ubp6 modulates protein turnover by removing ubiquitin chains of proteasome substrates and allosterically tuning proteasome activity, and its loss of function increases proteasome activity in cells (Hanna et al. 2006; Bashore et al. 2015). Accordingly, quantitative proteomics revealed that loss of *UBP6* lowers the levels of upregulated proteins and protein aggregates in the disomes (Oromendia et al. 2012, Dephoure et al. 2014). On the other hand, a genome-wide screen of the yeast deletion collection revealed that the deletion of *UBP3*, another deubiquitinating enzyme involved in the regulation of protein trafficking and required for stress response, lowers the fitness of most disomes (Dodgson et al. 2016). Therefore, suppressing or exacerbating proteotoxic stress represents viable strategies to improve the fitness of or target aneuploid cells. Importantly, a question that urges investigation is whether modulating protein degradation pathways in trisomy 21 significantly improves the fitness of these cells.

## An extra chromosome disrupts the morphology of the nucleus

**Fig. 4:**
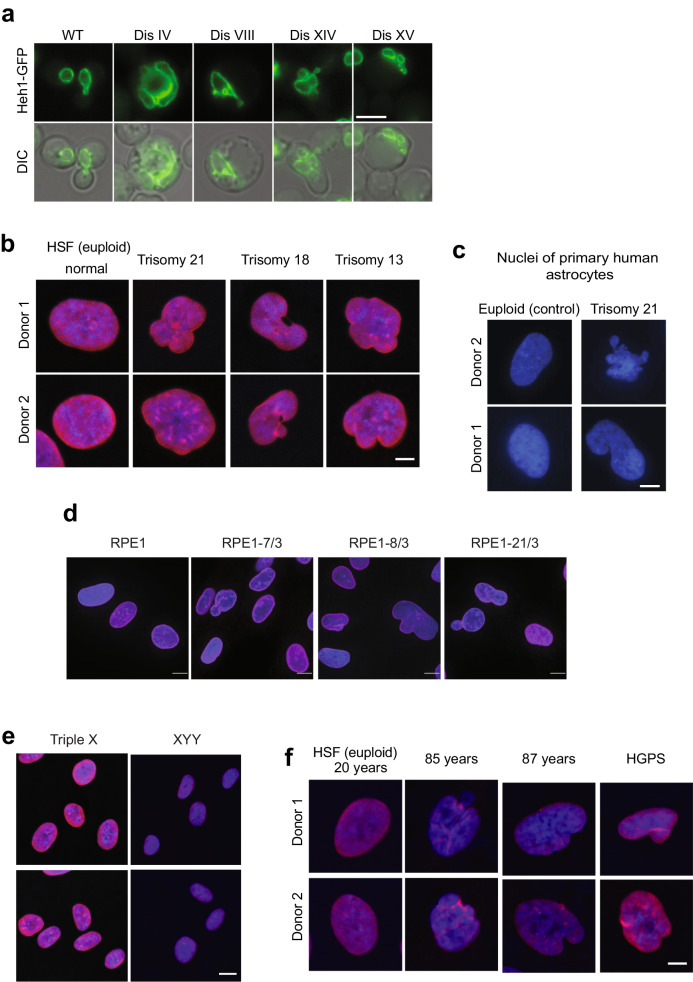
An extra chromosome disrupts the morphology of the nucleus. **a** Images of yeast cells expressing Heh1-GFP to mark the nuclear envelope shows that an extra chromosome disrupts the morphology of the nucleus. Differential interference contract is shown (DIC). Scale bar, 5 µm. **b** Immunofluorescence microscopy of lamin B1 in red and DNA in blue of human skin fibroblasts (HSFs). Scale bar, 2.5 µm. Up to 50% of the population of trisomic HSFs show abnormal shapes compared to euploid controls. **c** Images of primary human astrocytes from 2 euploid donors and 2 donors with Down syndrome. DNA is stained blue. Scale bar, 2.5 µm. **d** Immunofluorescence microscopy of lamin B1 in red and DNA in blue of RPE1 cells harboring extra chromosomes. Scale bar, 5 µm. **e** Immunofluorescence microscopy of lamin B1 in red and DNA in blue of HSFs from donors with triple X and XYY syndromes. Scale bar, 5 µm. **f** Immunofluorescence microscopy of lamin B1 in red and DNA in blue of HSFs from young, old donors, and donor with Hutchinson-Gilford progeria syndrome. Scale bar, 2.5 µm. Images adapted from Hwang et al. (Hwang et al. 2019)

Diploid yeast cells are double the volume of haploid cells as the cell volume is tightly coupled to the amount of DNA (Amodeo and Skotheim 2016). The amount of DNA influences nuclear volume, thus creating a link between nuclear and cell volumes, both regulated by poorly understood mechanisms (Cantwell and Nurse 2019). Consistent with the hypothesis that the amount of DNA influences cell volume, disomic yeast strains show increased cell volumes compared to the euploid control. These increases correlate with the size of the duplicated chromosome (Torres et al. 2007). Analysis of the nucleus of disomic yeast revealed that an extra chromosome also increases nuclear volume, thereby maintaining a constant cell-to-nuclear volume ratio (Hwang et al. 2019). Strikingly, live-cell microscopy revealed that while most disomic cells show increases in nuclear volumes and maintained a morphology typical of control cells, a significant part of the population of aneuploid yeasts showed abnormal nuclear morphologies (Fig. 4a) (Hwang et al. 2019). While euploid control yeast shows a homogeneous, primarily round nucleus, 5% to 50% of cells, depending on the disomic chromosome, show irregular nuclear morphologies. Phenotypic variability, a hallmark of aneuploid cells, explains the heterogeneity in nuclear morphology among genetically homogeneous populations of disomic yeasts (Beach et al. 2017). Furthermore, the degree by which the nucleus is affected in aneuploid yeast correlates with the number of protein-coding genes in the duplicated chromosome. These results indicate that harboring an extra chromosome requires increasing the volume of the nucleus, often disrupting the mechanisms that maintain its structural integrity.

Trisomic MEFs for chromosomes 1, 13, 16, or 19 also show increases in cell volumes proportional to the number of protein-coding genes in the extra chromosome (Williams et al. 2008). Yet, no volume changes have been reported in trisomic HSFs for chromosomes 13, 18, or 21. Nonetheless, analysis by immunofluorescence revealed that the nucleus of trisomic HSFs also shows irregular morphologies compared to euploid controls (Fig. 4b) (Hwang et al. 2019). Phenotypic variability is observed in trisomic HSFs regarding the abnormal nuclear morphologies, with up to 50% of the cells showing abnormalities that vary in severity. The abnormal nuclear morphologies of trisomy 21 HSFs were confirmed in 7 different cell lines obtained from donors ranging from newborn to 21 years old, indicating that the donor’s age did not cause this phenotype. Analysis of astrocytes isolated from Down syndrome individuals also shows irregular nuclear morphologies compared to euploid controls (Fig. 4c). Lastly, an extra copy of chromosomes 7, 8, or 21 causes abnormal nuclear shapes in RPE1 cells (Fig. 4d) (Hwang et al. 2019). These results indicate that aneuploidy disrupts nuclear morphology in yeast and several human cell types independent of the identity of the extra chromosome.

The mechanisms that cause changes in nuclear morphology in aneuploid cells are poorly understood (Zink et al. 2004; Fischer 2020). Interestingly, yeast cells harboring a YAC without protein-coding genes did not show similar nuclear defects. HSFs from donors with triple X or XYY syndromes show normal nuclear morphologies (Fig. 4e). These data suggest that an active chromosome and increased protein synthesis, not the mere presence of extra DNA, causes the nuclear phenotypes. One possibility is that together with the extra DNA, increases in mRNA content and protein levels associated with transcription of the extra chromosome increase the biomass within the nucleus. More molecules in the nucleus could alter the nucleoplasmic molarity causing physical stress in the nuclear membrane. It remains to be determined whether the nucleus of aneuploid cells accumulates more biomass or whether the biophysical properties of the nuclear membrane are affected by the presence of an extra autosome. Lamin proteins are a major determinant of the integrity and shape of the nuclear envelope in humans. Analysis of lamin levels by immunofluorescence, western blot, and mass spectrometry did not show changes in the trisomic HSFs. Since yeast does not encode lamin proteins, dysregulation of the nuclear lamins cannot account for a general mechanism for the abnormal nuclear morphologies in aneuploid cells. Furthermore, analysis of other factors associated with nuclear morphology, such as the levels of the tri-methylation of lysine 9 of histone H3 (H3K9triMe), an established marker of lamina-associated domains, did not correlate with altered nuclear morphology of trisomic HSF. Actin cables also show typical staining in trisomic HSF with abnormal nuclear morphology (Hwang et al. 2019).

Insight into what contributes to the nuclear morphology abnormalities in aneuploid cells comes from the analysis of mutations that improve the fitness of aneuploid yeast. In a selection screen, 2 out of 43 identified mutations improved the fitness of multiple disomes independent of the identity of the duplicated chromosome (Torres et al. 2010). As mentioned earlier, *ubp6∆* is one of them. While *upb6∆* improves the fitness of aneuploid cells by increasing proteasome activity, this mutation does not suppress the abnormal nuclear morphology of the disomes. The other mutation is *svf1∆*, a gene that regulates sphingolipid synthesis, implicating this class of lipids in the cellular response to aneuploidy (Hwang et al. 2017; Tang et al. 2017, Hwang et al. 2019). Sphingolipids, glycerophospholipids, and sterols (ergosterol in yeast and cholesterol in humans) are the three major structural lipids that constitute cellular membranes. Sphingolipids are made from the condensation reaction of serine and palmitoyl-CoA by serine palmitoyltransferase (SPT), a conserved enzyme localized in the nuclear envelope and endoplasmic reticulum. The product of SPT, 3-ketodihydrosphingosine, is rapidly metabolized to yield dihydroshingosine (DHS), which in yeast is hydroxylated to yield phytosphingosine. The products of SPT are collectively referred to as long-chain bases (LCBs, the base refers to the amine group coming from serine). In humans, the major forms of LCBs are DHS and sphingosine. LCBs are acylated by ceramide synthase to generate ceramides. Conservation between yeast and humans stops at ceramide, as ceramide is converted to complex sphingolipids, which are very different in these organisms (Dickson and Lester 2002; Hannun and Obeid 2008).

Although the precise function of *SVF1* is unknown, deletion of this gene leads to the accumulation of LCBs suggesting that these molecules play a role in the fitness of aneuploid cells (Brace et al. 2007; Hwang et al. 2017). A systematic genetic analysis in combination with drug treatments revealed independent strategies, all of which increased LCBs, to improve the fitness of almost all aneuploid yeast strains independent of karyotype (Hwang et al. 2017). Mutations or drugs that decreased LCBs amounts lowered the fitness of aneuploid yeast. One of the mutations that improved fitness in all disomes analyzed is the loss of ceramide synthase, *lag1∆*. Since ceramide synthase converts LCBs into ceramides, lower enzymatic activity leads to LCB accumulation. Quantitative mass spectrometry (MS) confirmed that *lag1∆* minimally affects the ceramide levels while LCBs increased by sevenfold in yeast cells (Hwang et al. 2019). Ceramide levels remain primarily unchanged due to the activity of *LAC1*, the other ceramide synthase paralog in yeast. Remarkably, *lag1∆* suppressed the nuclear defects of all the disomes irrespective of the extra chromosome’s identity. These results suggest that increases in LCB levels improve fitness in aneuploid cells by suppressing nuclear abnormalities. In supporting this hypothesis, quantifying sphingolipids in purified nuclei revealed that LCBs are enriched in the nuclear membrane of yeast cells and that the increases upon the loss of ceramide synthase accumulate in the nuclear membrane (Hwang et al. 2019). Notably, inhibition of SPT disrupts nuclear morphology in euploid cells indicating that LCBs constitute an essential structural component of the nuclear membrane.

Increasing the levels of LCBs also improves the nuclear defects of HSFs trisomic for chromosomes 21, 18, and 13 (Hwang et al. 2019). Whereas fumonisin B1, an inhibitor of ceramide synthase, minimally affects the nucleus of euploid controls, it significantly suppresses nuclear abnormalities in trisomic HSFs. Quantitative mass spectrometry revealed that fumonisin B1 leads to a 15-fold increase in LCB levels while ceramide levels minimally changed. Furthermore, fumonisin B1 also improves the proliferation rates of trisomic HSF, indicating that abnormal nuclear morphologies are associated with impaired proliferation. Targeting ceramide synthesis represents a novel strategy to suppress aneuploidy-driven phenotypes in human cells. Altogether, these results indicate that aneuploidy disrupts nuclear morphology and that dysregulation of the synthesis of LCBs enriched in the nuclear membrane accounts for this phenotype.

The physiological relevance of abnormal nuclear morphology in aneuploid cells is unknown; however, an irregular nucleus is associated with human disease and aging. In cancer, pathologists analyze clinical samples and use the degree of nuclear abnormalities to stage the grade of human tumors (Fischer 2020). As a degree of aneuploidy correlates with tumor progression, one possibility is that aneuploidy may be a driver of the pathophysiology of the nucleus in cancer. Interestingly, abnormal nuclear morphology is also a hallmark of aging as fibroblasts isolated from old individuals show abnormal nuclear morphologies similar to HSF trisomic for chromosome 21 (Scaffidi and Misteli 2006) (Fig. 4f). As the incidence of aneuploidy increases with aging, another possibility is that an abnormal number of chromosomes in old cells disrupt the morphology of the nucleus.

Insights into the aging process have come from studies of human diseases associated with premature aging as mutations in the lamin A/C gene LMNA cause the Hutchinson-Gilford progeria syndrome (HGPS) (De Sandre-Giovannoli et al. 2003). Mechanistically, silent mutations in LMNA affect splicing, leading to the expression of a dominant-negative truncated version of lamin named progerin. Progerin accumulates in the inner nuclear envelope and impacts its morphology and function. These results indicate that altered nuclear morphology is a hallmark of aging; however, the functional connection between an abnormal nucleus and aging is unknown. Remarkably fibroblasts with trisomy 21 show irregular nuclei reminiscent of cells from patients with HGPS (Fig. 4f). Based on these results, a possible hypothesis is that aneuploidy plays a causative role in the deterioration of cellular function during healthy aging and that this process is accelerated in individuals with Down syndrome. Although the genetic bases of progeroid syndromes are unrelated to Down syndrome, several clinical symptoms are shared between these two conditions. These include growth retardation, skeletal and craniofacial anomalies, heart defects, abnormal skin, low muscle tone, gastrointestinal issues, and poor immunity.

## Aneuploidy increases metabolic demand for serine and sphingolipids

**Fig. 5:**
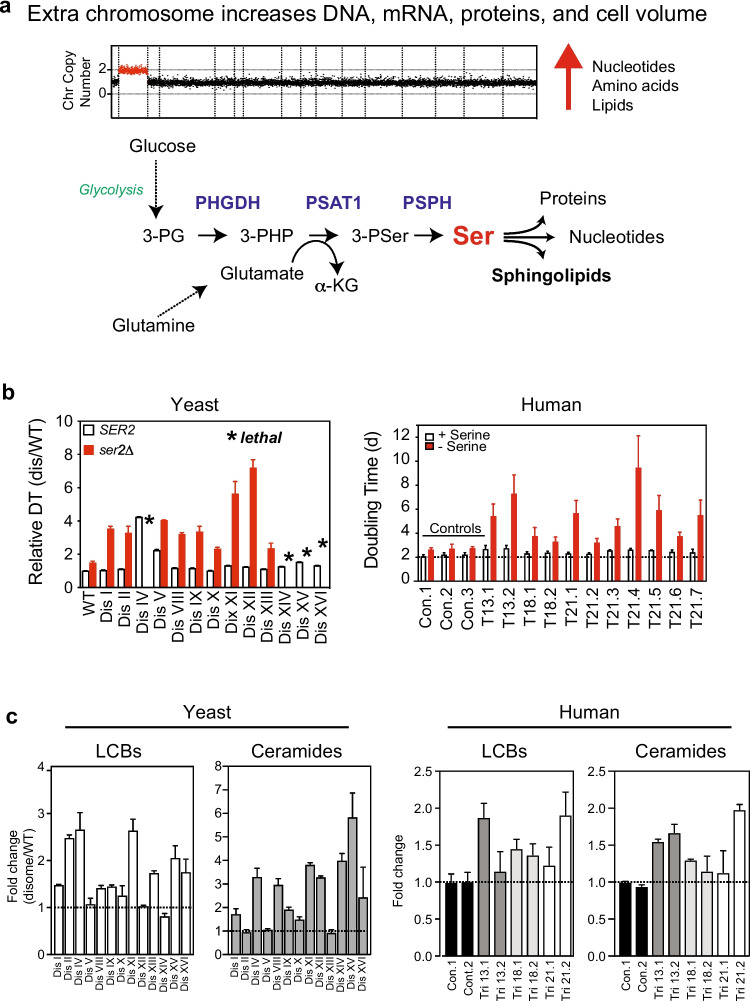
Aneuploid cells rely on increased serine synthesis to survive. **a** A gain of an extra chromosome increases the demand for biomass synthesis including nucleic acids, amino acids, and lipid synthesis. The biosynthesis of serine is required for the survival of aneuploid cells because serine is used to make other amino acids and proteins, nucleotides, and lipids. The de novo biosynthesis of serine requires increased utilization of glucose and glutamine. 3-PG, 3-phoshoglycerate; 3-PHP, 3-phosphopyruvate; 3-PSer, 3-phosphoserine; alpha-KG, alpha-ketoglutarate; PHGDH, 3-phosphoglycerate dehydrogenase; PSAT1, phosphoserine transaminase 1; PSPH, phosphoserine phosphatase. **b** In yeast, deletion of *SER2* (PSPH) hampers the proliferation of wild type cells grown in minimal media containing 1 mM serine. Disomic strains harboring the same deletion grow extremely poorly in minimal media containing 1 mM serine. Asterisk (*) indicates loss of *SER2* causes is lethal in disomes IV, XIV, XV, and XVI. Data adapted from Hwang et al. (Hwang et al. 2017). HSFs trisomic for chromosomes 13, 18, or 21 are sensitive to serine depletion from the growth media. Dis, disome; Con.1, control 1; T13.1, trisomy 13 donor 1. **c** Quantitative lipidomics shows that the levels of LCBs and ceramides are increased in aneuploid yeast and human cells. Data obtained from Hwang et al. (Hwang et al. 2017, Hwang et al. 2021)

Gaining an extra chromosome is accompanied by increases in DNA, RNA, and protein synthesis. Increases in cell and nuclear volume imply more membrane surface area that must be coupled with increased lipid synthesis. In all, biosynthesis of the building blocks of the cell, including nucleotides, amino acids, and lipids, must be affected upon gaining an extra chromosome (Fig. 5a). Consistent with the hypothesis that an extra chromosome increased metabolic demands, yeast disomes utilized more glucose than euploid control, while trisomic MEFs used more glutamine (Torres et al. 2007; Williams et al. 2008).

The biosynthesis of the amino acid serine has emerged as one pathway required for the proliferation of aneuploid cells (Fig. 5a). In yeast, quantitative proteomics revealed that the disomes upregulate the protein levels of the phosphoserine phosphatase Ser2, a conserved enzyme required for the last step in serine biosynthesis from glucose (Dephoure et al. 2014, Hwang et al. 2017). Remarkably, the deletion of *SER2* significantly hampered the proliferation of all disomes relative to the euploid control and even caused lethality to several disomes that harbor large chromosomes (Fig. 5b). Serine is also essential for the growth of aneuploid human cells since serine depletion from the growth medium causes a dramatic effect in the proliferation of HSFs trisomic for chromosomes 21, 18, or 13 compared to euploid controls (Fig. 5b) (Hwang et al. 2021). These results support the hypothesis that cells with an extra chromosome rely on increased serine synthesis to proliferate. Indeed, serine biosynthesis has been implicated in the survival of aneuploid tumor cells (Locasale 2013). Serine is a key amino acid utilized to synthesize other amino acids and for lipid synthesis, including sphingolipids and phosphatidylserine. Serine also fuels the one-carbon cycle coupled to nucleotide biosynthesis. The mechanisms by which the cell senses the increased demand for biomass production and upregulates serine synthesis are not understood. Interestingly, Ser2 protein in aneuploid yeast is upregulated by posttranscriptional mechanisms, as the transcript levels are unaffected.

One of the pathways strictly dependent on serine is the synthesis of sphingolipids. Global lipidome analysis using quantitative mass spectrometry of disomic yeast strains revealed that LCBs and ceramides are elevated compared to euploid control (Fig. 5c) (Hwang et al. 2017). Importantly, glycerophospholipids and complex sphingolipids levels are not significantly affected in aneuploid yeasts. Furthermore, quantitative lipidomics revealed that the increases of LCB accumulate at the nuclear membrane supporting the hypothesis that increasing LCB levels are driven by changes in the lipid requirements to maintain the nuclear integrity due to the presence of the extra chromosomes. Indeed, aneuploid yeast cells are hypersensitive to myriocin, a drug that specifically inhibits SPT, suggesting that LCB level increases are required for viability. Yet, as mentioned earlier, further increases by mutation of genes that regulate LCB biosynthesis significantly improve the fitness of the disomes. LCB and ceramides are also elevated in trisomic MEFs for chromosomes 1, 13, 16, and 19 and HSF trisomic for chromosomes 21, 18, and 13 (Fig. 5c) (Tang et al. 2017, Hwang et al. 2019). These results indicate that an increased demand for sphingolipids biosynthesis is a hallmark of aneuploid cells in yeast and human cells.

## Concluding remarks

Increased activity of genes on chromosome 21 is associated with several pathologies in Down syndrome (Antonarakis et al. 2020). Studies of the consequences of aneuploidy on cell physiology suggest that in addition to increased gene activity, the general disruption of cellular homeostasis associated with aneuploidy may cause pathophysiologies in Down syndrome. Lower cellular fitness and viability may account for the hypocellularity observed in many tissues with trisomy 21 that also display smaller than average size. Remarkably, an unbiased metabolic profile of plasma samples of hundreds of individuals revealed that among the metabolites affected by Down syndrome are serine and sphingosine-1-phosphate (Powers et al. 2019). The potential for clinical intervention to improve the fitness of trisomy 21 cells in vivo by supplementing serine and sphingolipids to improve cellular viability represents an exciting area of future research.

The role of aneuploidy in cancer is complicated, as chromosomal instability causes aneuploidy, and aneuploid cells show increased genomic instability (Sheltzer et al. 2011; Passerini et al. 2016). While some studies propose that aneuploidy inhibits tumor growth (Sheltzer et al. 2017), other studies suggest that gaining a chromosome can drive tumorigenesis (Girish et al. 2023). The critical challenge is determining the molecular basis for increased chromosomal instability in aneuploid cells. Whether proteotoxic stress, abnormal nuclear structure, or altered metabolism drives this phenotype remains unknown. Furthermore, deciphering what genomic alterations are acquired during tumor evolution to alleviate the harmful effects of aneuploidy could lead to identifying specific pathways that could be targeted to kill aneuploid cells. This review’s scope was limited to comparing yeast and human cells to highlight conserved cellular processes affected by aneuploidy. It is important to emphasize that human aneuploidy has been implicated in immune responses, and conflicting conclusions exist regarding whether aneuploid cells inhibit or promote an immune response (Davoli et al. 2017, Santaguida et al. 2017). The effects of aneuploidy on human immunity is an exciting area of research that points to the limitations of model organisms such as yeast to determine the role of aneuploidy in human disease.

## Data Availability

All the data has been previously published and it is available from the references cited.
